# Dental Anæsthetics, Etc.

**Published:** 1893-05

**Authors:** 


### Dental Anæsthetics, Etc.

EDITOR MEDICAL WORLD :—W. H. DeKay asks for the formula of the dental anæsthetic known as "Otandunder." I made an analysis of a similar compound called anæsthetine, and found it to be a four per cent. solution of cocaine, boracic acid, creosote and glycerine. I have used for some time a local anæsthetic for the extraction of teeth, which always acts promptly and is prepared as follows:

R. Olei Gaultheriæ..............drams ij
Chloroformi ....................dram j
Ether sulph ..... ..............dram j
Chloral hydrat..............drams ij
Olei caryophilli...............drams iv
Alcoholis...................ounces iss

M. Sig.—Apply with cotton, pressed upon each side of the tooth.

